# Identifying Regenerated Saplings by Stratifying Forest Overstory Using Airborne LiDAR Data

**DOI:** 10.34133/plantphenomics.0145

**Published:** 2024-02-08

**Authors:** Liming Du, Yong Pang

**Affiliations:** ^1^ Institute of Forest Resource Information Techniques, Chinese Academy of Forestry, Beijing 100091, China.; ^2^ Key Laboratory of Forestry Remote Sensing and Information System, National Forestry and Grassland Administration, Beijing 100091, China.

## Abstract

Identifying the spatiotemporal distributions and phenotypic characteristics of understory saplings is beneficial in exploring the internal mechanisms of plant regeneration and providing technical assistances for continues cover forest management. However, it is challenging to detect the understory saplings using 2-dimensional (2D) spectral information produced by conventional optical remotely sensed data. This study proposed an automatic method to detect the regenerated understory saplings based on the 3D structural information from aerial laser scanning (ALS) data. By delineating individual tree crown using the improved spectral clustering algorithm, we successfully removed the overstory canopy and associated trunk points. Then, individual understory saplings were segmented using an adaptive-mean-shift-based clustering algorithm. This method was tested in an experimental forest farm of North China. Our results showed that the detection rates of understory saplings ranged from 94.41% to 152.78%, and the matching rates increased from 62.59% to 95.65% as canopy closure went down. The ALS-based sapling heights well captured the variations of field measurements [*R*^2^ = 0.71, *N* = 3,241, root mean square error (RMSE) = 0.26 m, *P* < 0.01] and terrestrial laser scanning (TLS)-based measurements (*R*^2^ = 0.78, *N* =443, RMSE = 0.23 m, *P* < 0.01). The ALS-based sapling crown width was comparable with TLS-based measurements (*R*^2^ = 0.64, *N* = 443, RMSE = 0.24 m). This study provides a solution for the quantification of understory saplings, which can be used to improve forest ecosystem resilence through regulating the dynamics of forest gaps to better utilize light resources.

## Introduction

Regenerated saplings are crucial for an ecologically resilient forest to maintain diverse and productive ecosystem functions. Moreover, it is beneficial to sustainable forest management and continuous forest coverage [1]. Both the natural regeneration and planted saplings are commonly used in silviculture treatments. Clarifying the spatial distribution and phenotypic parameters of understory saplings is of great significance for studying the influence of canopy structural characteristics and the dynamic changes of the light regime under the forest on the growth of saplings and further exploring the internal mechanisms of regeneration and succession of plants under the forest [2,3]. Meanwhile, the phenotypic parameters such as tree height, diameter at breast height (DBH), and crown width are critical for sapling breeding and forest management in terms of genetic material selection [4]. However, the acquisition of forest phenotypic parameters is a challenge because trees are long-lived with a wide range of growth and variable shape, which requires long-term and high-frequency monitoring [5]. Traditional methods of manually measuring understory saplings to obtain phenotypic parameters in the field are destructive, time-consuming, and laborious. Active light detection and ranging (LiDAR) can penetrate the forest canopy through forest gaps and obtain a high precision 3-dimensional (3D) point clouds from the top of the forest to the ground [6], which provides new light for the acquisition of forest phenotypic parameters.

Compared with terrestrial laser scanning (TLS) or unmanned-aerial-vehicle-based laser scanning that is more suitable for small scale forest monitoring [7,8] and spaceborne LiDAR with sparse laser footprints [9,10], aerial laser scanning (ALS) simultaneously takes into account the measurement scale and point density, which can provide a wide range of phenotypic parameters of regenerated saplings, including tree height and crown width [11–13].

Some scholars have used ALS point cloud data to detect the tree height and distribution information of regenerated forests. For example, Latifi et al. [14] and Venier et al. [15] have collected high-density ALS point clouds and estimated understory coverage using different models and LiDAR metrics. Imangholiloo et al. [16] have evaluated the ability of multispectral airborne laser scanning data to characterize sapling stands under leaf-off and leaf-on conditions and estimated the stem density and average tree height. Næsset and Bjerknes [17] and Ørka et al. [18] have used ALS-based metrics to estimate tree density and mean height of trees during regeneration. In general, the existing methods rarely pay attention to the estimation of the phenotypic parameters of understory saplings, which may be limited by the downward-looking scanning mode of ALS and the precision of its point cloud (centimeter magnitude) [19]. The variation of phenotypic parameters in understory saplings is greatly affected by canopy gap characteristics [20]. Enough gaps not only provide growth space for the regeneration of understory saplings but also lead to lower stem densities of the upper mature trees, which are easier to be penetrated by ALS [21]. With increasing point density, ALS technology has the potential to describe the internal 3D structure of regenerated forests, opening new avenues for estimating tree height and crown width of understory saplings.

Over the past decades, numerous studies have been carried out to develop rapid and nondestructive methods for extracting phenotypic parameters such as individual tree height and crown width based on ALS data [22,23]. Two classical and frequently used methods include raster-based algorithms that utilize point clouds indirectly and algorithms that directly utilize full 3D point clouds [24]. Raster-based methods perform preprocessing such as filtering, normalization, interpolation, and smoothing on the raw point cloud and convert them into a canopy height model (CHM). On this basis, traditional tree detection and delineation methods (such as template matching [25], region-growing [26], valley-following [27], and watershed segmentation [28]) were used to extract tree heights and crown width of individual trees. This method has the benefits of being well developed, having high segmentation efficiency, being simple to use and improve, and so on. However, information loss is easily caused in the process of projecting 3D point clouds to 2D raster, and the parameters of individual trees oppressed by dominant trees are difficult to be accurately detected [29]. Meanwhile, these methods are susceptible to noise and are prone to oversegmentation and undersegmentation [30]. Point-cloud-based methods using the whole dataset were expected to yield more accurate tree heights and crown width of individual trees, especially for those stands with complex vertical structures [31]. Two classic point-based methods are *k*-means clustering techniques [32] and voxel-based individual tree segmentation methods [33]. The *k*-means method finds *k* local maxima in space as seeds and aggregates the points that are closest to them [34]. This method has low computational complexity, but the segmentation results are strongly affected by the accuracy of the detected local maximum points. The voxel-based method constructs voxels with different resolutions based on point clouds and further extracts individual trees through graph cutting or clustering [35].

Although individual tree segmentation algorithms have come a long way, no one has yet found a way to use them to separate understory saplings and extract their phenotypic parameters. The goal of this paper is to figure out how to divide understory saplings into groups based on removing overstory mature trees accurately. High-density laser point clouds were firstly obtained by fusing the data collected from multiple flight lines at different observation positions above the study area. Then, upper mature trees were segmented through the Nyström-based spectral clustering (NSC) algorithm. A fine postprocessing method was proposed to overcome the oversegmentation and undersegmentation problems in the results and further improve the position accuracy of upper mature trees. Furthermore, on the basis of removing the points of mature trees in the upper layer, the local adaptive mean shift algorithm was developed to delineate the understory saplings. The method proposed in this paper can automatically realize the segmentation of understory saplings within a large range, obtain their phenotypic parameters such as tree height and crown width, and provide technical support for studying the growth status of saplings and forest management planning.

## Materials and Methods

### Study area

The study was conducted at the Saihanba Forest Farm in Hebei province, which is located at the junction of the southern edge of the Inner Mongolia Plateau and the northern Hebei mountains (42°18′57″ to 42°34′54″N, 117°06′32″ to 117°30′23″E). The forest farm is an important plantation demonstration forest farm in North China. The elevation of the area varies from 1,440 to 1,910 m above sea level. The terrain is gentle slope with the mean value of approximately 8.84°. The study area has a cold temperate continental monsoon climate, with an average annual temperature of −1.4 °C and an average annual rainfall of 453.6 mm. The dominant tree species of overstory is *Larix principis-rupprechtii*. The *Picea asperata* saplings were planted in the *Larix* gaps over large areas with nearly equal spacing between rows and columns. The purpose of these planted saplings is to improve the resilient status from monoculture to mixed species, make full use of the land resources from 1 layer to 2 layer stands, promote ecological and economic benefits, and take into account the advantages of mixed forest (e.g., enhancing the stability of the forest stands and reducing pests and diseases, etc.).

### LiDAR and field data acquisition

#### High-density ALS data

ALS data of the Saihanba Forest Farm were collected in September 2018 using the LiDAR, CCD, and hyperspectral airborne observation system of the Chinese Academy of Forestry (CAF-LiCHy), which was mounted on a helicopter and flown at 500 m above ground level with the mean strip overlap rate of 58.6% [36]. The Rigel LMS-Q680i laser scanner with the wavelength of 1,550 nm and the beam divergence of 0.5 mrad was integrated in this system. The laser repetition pulse rate of the scanner was between 80 to 400 kHz with a pulse length of 3 ns. Furthermore, 21 flight lines with multiple flight directions were designed to obtain data. The dense point cloud data were realized through the relative matching of different flight lines. The final matching error of multiple flight line data is less than 10 cm, and the average point density is 243 points/m^2^.

#### TLS data

**Fig. 1.**
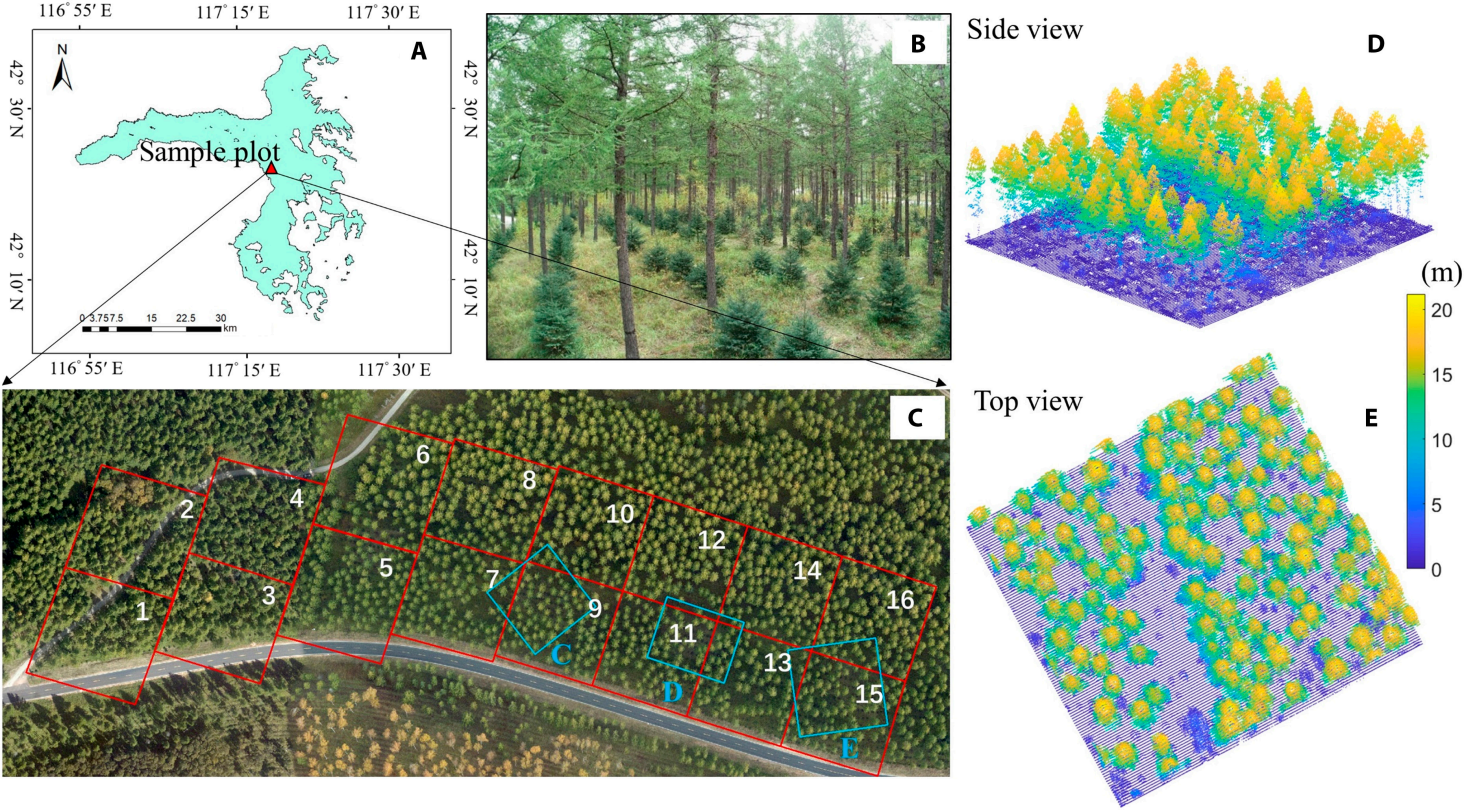
Distribution of the study area, ALS point clouds, and approximate location of the reference data used in this study. (A) The location of the sample plot. (B) The actual scene of understory saplings within the image obtained through unmanned aerial vehicle. (C) The orthoimage of the sample plot, which was divided into 15 subplots with the same size and exhibited as red squares. The blue polygons superimposed on red squares indicate the ranges where TLS data were collected. (D) Side view and (E) top view of ALS point clouds of the study area, including the mature trees and understory saplings.

Three regions with different sizes were arranged within the sample plot (as shown in Fig. 1) for TLS data collection in September 2018, which was synchronous with ALS data collection. The Trimble TX8 3D laser scanner with the laser wavelength of 500 nm was used. The ranging noise of the laser scanner is less than 2 mm, and the maximum measurement distance is 120 m. In the process of data acquisition, each region was scanned with one station at each corner stake and one station at the plot center corner stake that has been positioned with Global Navigation Satellite System (GNSS). Meanwhile, 10 round target balls were evenly placed in each sample plot for multistation data registration. After data acquisition, the TLS stations of each sample plot were registered using the Trimble RealWorks software and clipped depending on the boundaries. Then, the data were denoised, classified, and elevation-normalized. The sapling points were selected from the data and segmented through the watershed algorithm in the MATLAB toolbox. Then, the points of terminal leader shoot were removed from each sapling according to the variation of vertical profile [37].

#### Field data

A total of 3,963 spruce saplings planted at the lower layer of *Larch* in the sample plot with the total area of 4.69 ha were measured in detail. Field work over these trees was conducted in August 2017. The locations of the plot center and corners were measured using a GNSS receiver, which was corrected with differential signals obtained from a reference receiver located in an open space near the corresponding plot. Stem positions of saplings were obtained by real-time kinematic GNSS system through placing positioning equipment on the same side of the saplings. To improve the positioning accuracy, all measured coordinates were calibrated to the center of the saplings as a whole through the human-machine interactive spatial calibration method. Sapling heights were measured using a height measuring rod. The sample plot was further divided into 15 subplots with the same dimensions of about 50 m × 50 m and used to verify the proposed method (Fig. 1).

### Methods

#### Method description

**Fig. 2.**
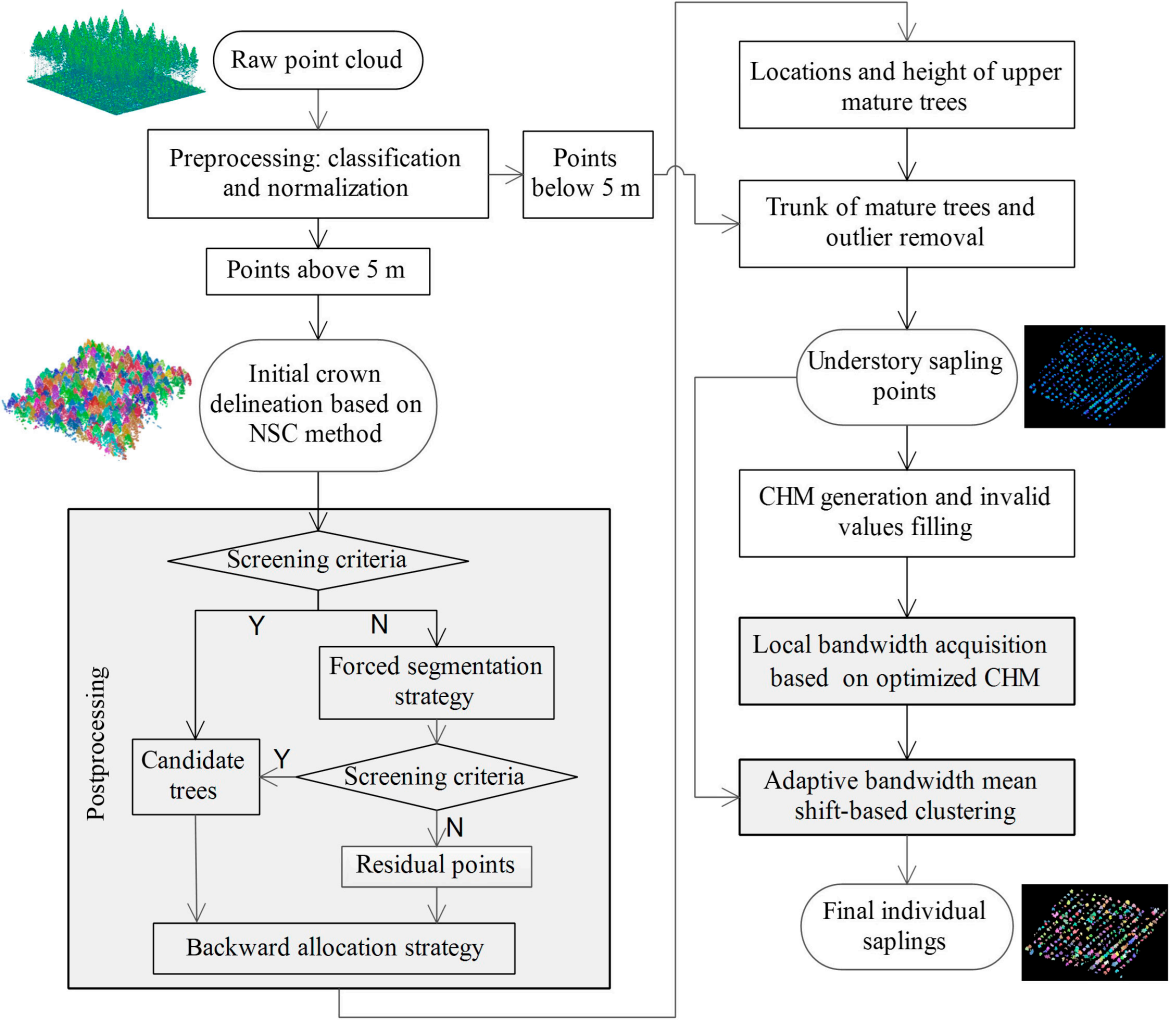
Flowchart of methods used in this study. The left part of the flowchart shows the segmentation process of the overstory mature trees, and the right part of the flowchart indicates the segmentation process of the understory saplings. The main algorithm innovation parts of this study are displayed with gray background.

ALS technology with high density points can penetrate overstory through forest gaps and detect the 3D structure of understory saplings. However, the interception of laser signals by the forest canopy and the close distance between the trunks of mature trees and saplings brought great interference to the extraction of sapling points. Therefore, the laser point clouds of all mature trees (including points of crowns, trunk, and branch) were first removed through accurate individual tree segmentation for canopy. The NSC method was used to delineate the tree crowns. To further improve the accuracy of the segmentation results, 2 postprocessing strategies were proposed and used to obtain the accurate position and phenotypic parameters of each upper mature tree. These tree position and height values of mature trees were further used to completely remove the overstory point clouds. The remaining points were processed to remove the outliers and utilized to generate the CHM. In addition, adaptive kernel function bandwidth of the mean shift algorithm was determined on the basis of the initial positions and crown width of saplings detected by CHM. Finally, saplings were detected from 3D point clouds. The flowchart of methods used in this study was described in Fig. 2. The left part indicates the processing workflow of the upper mature trees and the right part elaborates the processing workflow of understory saplings. The mature algorithms (such as cloth simulation filtering, alpha shape, Douglas–Peucker, region growing algorithm, etc.) used in this study were all implemented through the MATLAB toolbox, and default parameters were used in these algorithms, if not specified below.

#### Fine normalization of point clouds

To decrease the impact of terrain on canopy delineation and sapling detection, the raw point clouds were classified and normalized. The cloth simulation filtering method was first used to separate the ground and nonground points [38]. However, laser signals of ALS technology were difficult to reach the ground under the forest and covered by dense shrub and grass even if the density of point cloud was very high. If the points of shrub or grass covered on the ground were misclassified as ground points, the normalized point clouds will lead to the underestimation of tree height, which will have a more serious impact on the saplings than mature trees. Therefore, the classified ground points were further divided into several regular voxels through increasing the *x* and *y* coordinates from the minimum *x* and *y* points in a certain step ∆ ( based on extensive analyses of the data used in this study, ∆ was commonly set to 0.5 m). In each voxel, the lowest point was detected and considered as a true ground point, and other classified ground points were considered as pseudo-ground points. Finally, the classified nonground points were further normalized on the basis of their closest true ground points in horizontal direction, and the elevation values of pseudo-ground points were still assigned to 0 m to reduce their interference to the sapling extraction. The specific preprocessing process was intuitively exhibited through Fig. S1.

#### Segmentation of the overstory mature trees

1. Initial canopy delineation using NSC method. According to the planting years, growth rate, and field measurements of overstory *L. principis-rupprechtii* and understory *P. asperata* saplings, the heights of all saplings do not exceed 3.5 m, and the heights of mature trees are all above 10 m. There is height difference between the overstory mature trees and understory saplings. Therefore, this paper set 5 m as the height threshold for further point cloud classification. The points above 5 m were selected from the normalized point clouds for canopy delineation, which can reduce the computational pressure of the segmentation algorithm. The NSC method was utilized to complete the segmentation process [31]. Although this algorithm obtained relatively satisfactory segmentation results, the problem of undersegmentation and oversegmentation still existed because of the intersection of tree crowns and branches in actual forest environment or nonadaptability of segmentation parameters. These errors led to inaccurate extraction of mature tree positions, which influenced the detection accuracy of saplings indirectly.
2. Postprocessing method of the NSC (NSCP) segmentation results. Some scholars noted that the crown of coniferous trees can be expressed as round or nearly round [39–41]. However, individual trees with segmentation errors usually show specificity in tree height, crown width, crown shape, number or density of tree points, etc. [24]. Therefore, we first used strict control factors to select all individual trees that may have segmentation errors. The segmented trees meeting the following 4 conditions simultaneously will be classified as candidate individual trees. Otherwise, they will be regarded as trees containing segmentation errors. Figure S2 displayed the filtering results of a portion of the NSC segmentation result.
  ① Tree height was higher than 5 m;
  ② The ratio of short side and long side of the minimum bounding rectangle of a tree crown *i* was less than 1.3 and greater than 0.7.
  ③ Tree centroid *C_i_* was within the range of tree crown.
  $$C_{i}∼\left(X_{\text{mean}}^{i},Y_{\text{mean}}^{i},Z_{\text{mean}}^{i}\right)$$ (1)
  ④ The number of tree points was higher than *num*_*mean*/2, where *num*_*mean* was the average point number of the candidate trees, its initial value can be obtained by randomly selecting samples from the segmentation results, and later change with the increase of candidate trees. Due to the fact that the mature trees in the study area had same age, similar site condition and management treatments, they showed similar external structural characteristics. Therefore, this was a loose rule that is used to screen out those segmentation results with only few points and obviously cannot form an individual tree.

**Fig. 3.**
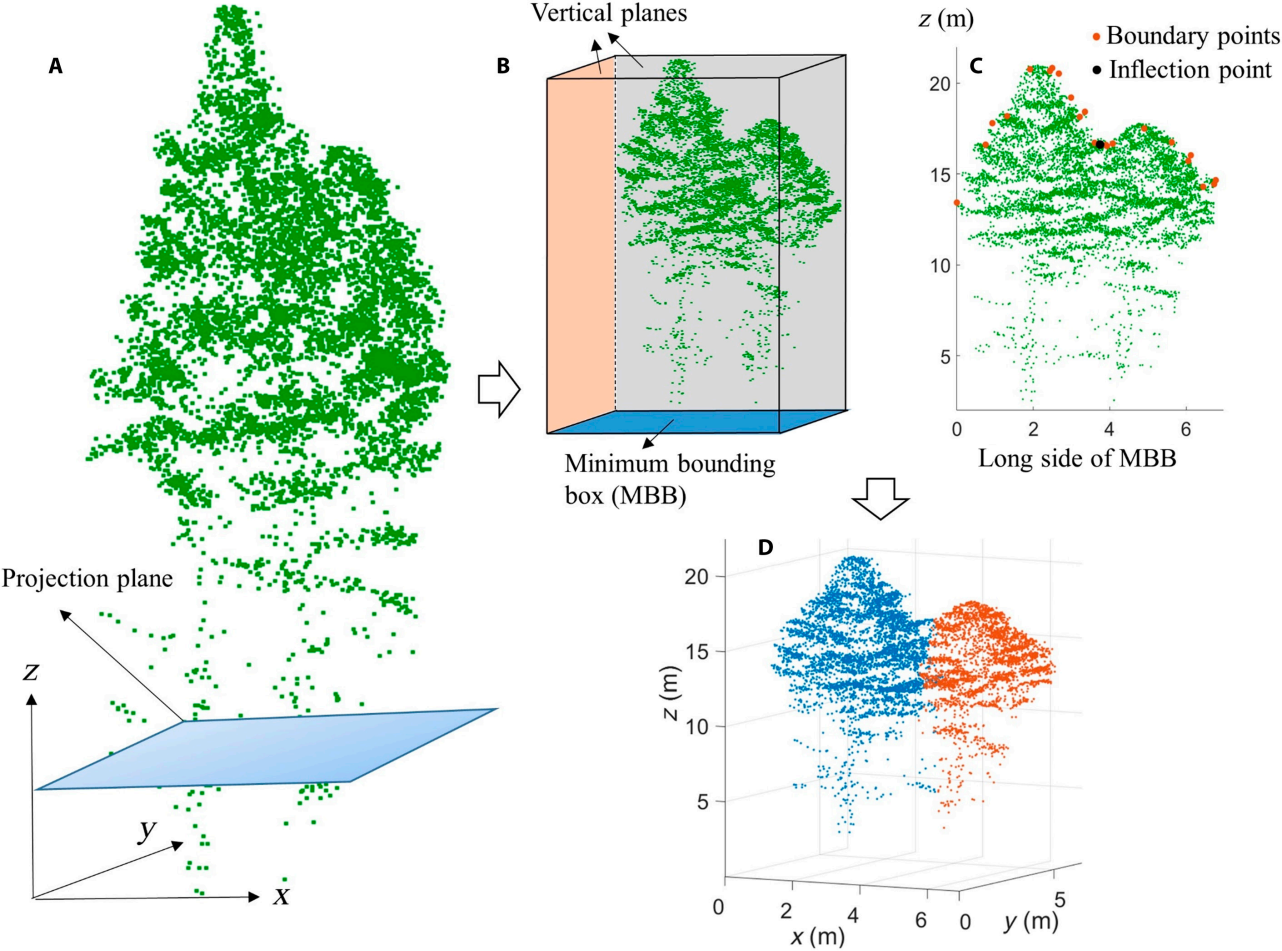
Postprocessing strategies for oversegmentation and undersegmentation trees. (A) Undersegmented tree and the projection plane. (B) Construct vertical planes using the minimum bounding box algorithm. (C) Inflection point detection. (D) Postprocessing result of the trees.

On this basis, forced segmentation strategy was further adopted to delineate the trees containing segmentation errors. As shown in Fig. 3, there was a undersegmentation error. To address this issue, the forced segmentation strategy involves the following steps. First, all the 3D tree point clouds including segmentation errors are projected to the XOY plane, and the minimum bounding box of each tree is calculated to obtain long and short sides of the tree crown. The planes passing through the sides and perpendicular to the ground constitute vertical facades (Fig. 3A and B). Second, 3D point clouds of the tree are projected to the 2 vertical planes respectively, and the projected boundary points of the tree crown are detected through the alpha shape algorithm (Fig. 3C) [42]. Then, the Douglas–Peucker algorithm [43] is used to simplify the projected boundary points and obtain possible inflection points. In this study, 5 times the average distance between adjacent points was set as the threshold condition of this algorithm. The final inflection point is obtained through taking the derivative of these possible inflection points. If no inflection point is detected in both projection directions, the tree is directly divided into the residual point set. Otherwise, the tree points are forcibly cut using the plane that passes through the inflection points and orthogonal to the corresponding projected facade (Fig. 3D). In practical situations, undersegmentation and oversegmentation errors often occur simultaneously, which can be further solved using the above algorithm. However, the trees with only oversegmentation error will be divided into several subtrees. In this case, inflection points cannot be detected since there are no multiple crowns in a subtree and these subtrees will be directly divided into the residual point set. Finally, conditions ① to ④ are further used to filter each tree, and those satisfying the conditions are classified as candidate individual trees; otherwise, they are divided into the residual point set.

**Fig. 4.**
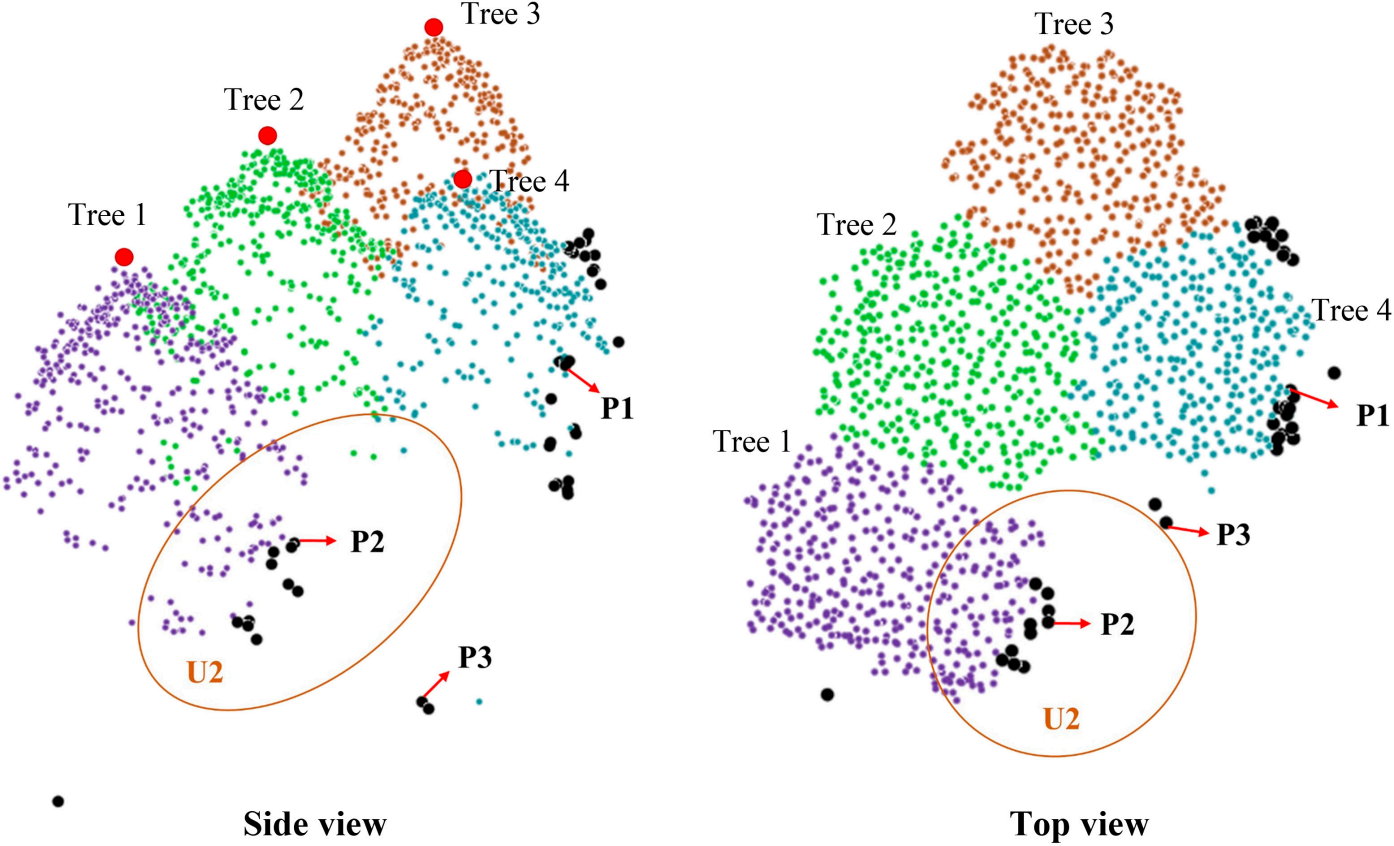
Backward allocation strategy of the NSCP method. Tree 1 to tree 4 with colored points indicate the candidate trees. The black points (including P1, P2, and P3) belong to the residual point set should be secondary distributed. The points of candidate trees in the orange circle U2 construct a local point set of P2.

In this paper, the backward allocation strategy was adopted to realize the secondary distribution of the points in the residual point set based on the candidate individual trees. Figure 4 is a simple schematic diagram used to explain the backward allocation strategy. In the figure, tree 1 to tree 4 with colored points are candidate individual trees, and the black points belong to the residual point set. First, the plane distances between each black point in the residual point set and all candidate individual trees are calculated. If there is a candidate tree whose plane distance from a black point is minimum and less than the crown width, the black point is directly merged with it. For instance, P1 can directly merge with tree 4. For black points that cannot directly merge with candidate trees, their local point sets are respectively constructed on the basis of the discrete points of the candidate trees. Specifically, when the plane distance of a discrete point and a black point is less than the threshold (the value of threshold was defined as the average crown radius of the nearest 3 trees of the black point), the discrete point is belong to the local point set of the black point. Then, the probability that each black point belongs to each candidate tree is judged by the following distance criterion.

• If the discrete points in the local point set U of a black point belong to one or more candidate trees, the number of the discrete points of each candidate tree in U is counted. The black point is finally merged into the candidate tree that accounts for the highest proportion of the point set U and has a proportion of more than 50%.
• Otherwise, the black point is directly judged as a noise point.

In Fig. 4, tree 1 is the candidate tree with the smallest plane distance from point P2, but this distance is still greater than the crown radius of tree 1. Therefore, the point set U2 of P2 is constructed on the basis of the candidate trees. According to the distance criterion, P2 is still assigned to tree 1. In contrast, P3 is judged as a noise point based on the above criterion. Finally, the structural parameters of each individual tree are further updated after the NSC segmentation results are postprocessed through accurate postprocessing algorithm.

#### Understory sapling detection and delineation

**Fig. 5.**
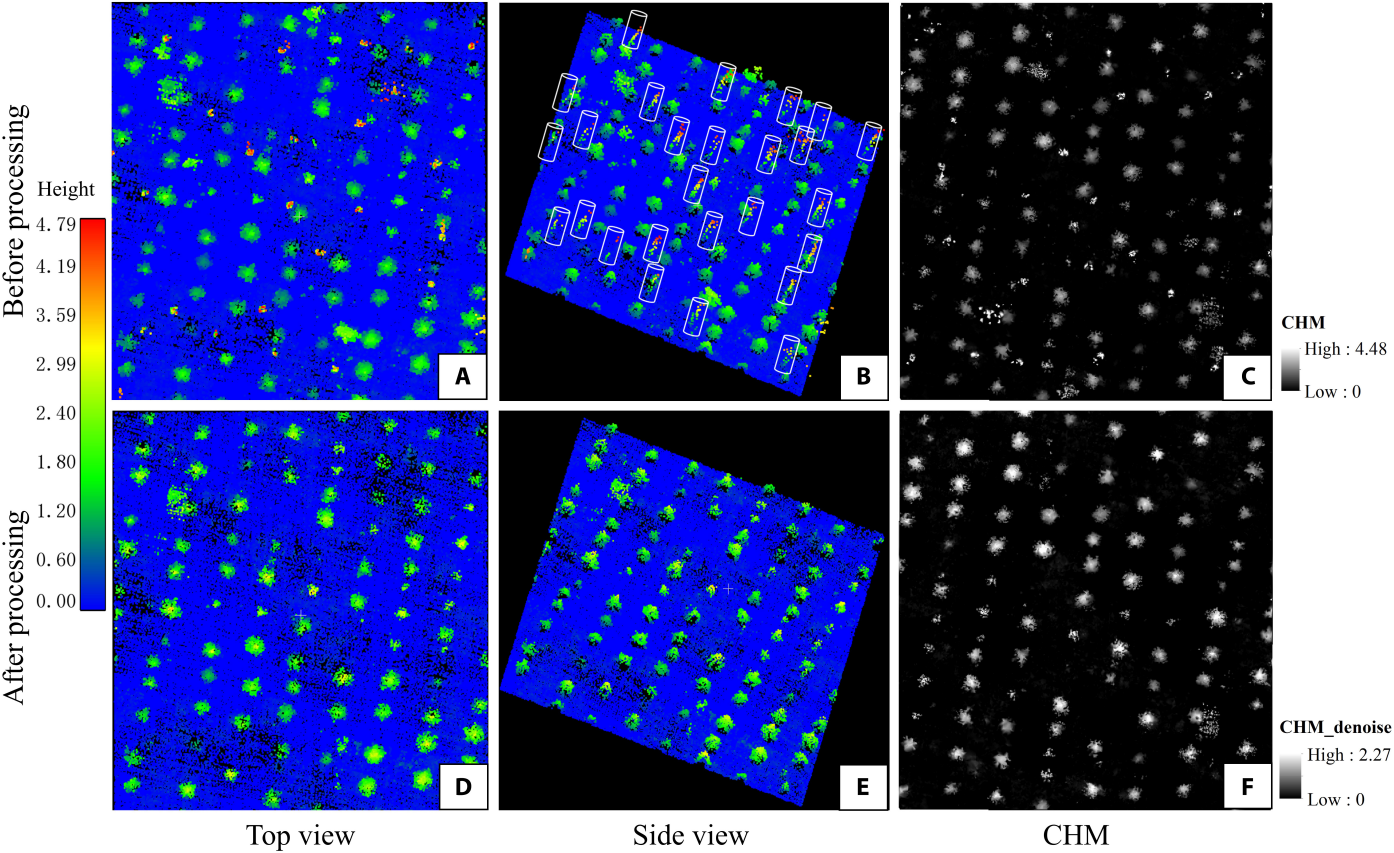
Overstory point clouds removal and sapling CHM generation. The subgraphs in each column indicate the front view, side view of sapling point clouds, and the CHM, respectively. (A to C) The subgraphs in the first row indicate the data that include the trunk points of mature trees, while (D to F) the subgraphs in the second row indicate the results that the trunk points of mature trees were removed. The cylinders in subgraph (B) represent the buffer zones obtained on the basis of the position and height of mature trees, which were used to remove the trunk points of the mature trees mixed in the saplings.

1. Remove the trunk points of mature trees. Sapling segmentation was carried out on the basis of the normalized high-density point clouds below 5 m. To remove the trunk points of mature trees interspersed in the saplings (Fig. 5A), the buffer zones were set according to the calculated stem coordinates and estimated DBH of the mature trees. One hundred mature trees were randomly selected from field measured data and used to establish the linear model between DBH and height. Then, the DBH of each mature tree was estimated on the basis of the tree height obtained from ALS data. Finally, 1.5 times the calculated DBH was used as the buffer zone value of each mature tree, as shown in Fig. 5B.

Although the trunk point clouds of upper mature trees were basically removed through the method above, some discrete points still left because of the existing shrubs and grass under the forest, inaccurate trunk position caused by crown inclination, etc. These discrete points would cause great interference to the extraction of saplings. Therefore, the outliers were further removed by the open source LASTools software. Through creating the cell of size of 0.3 by 0.3 by 0.3 units, the lasnoise function was used to remove isolated points not belonging to saplings, as shown in Fig. 5D and E. Finally, the CHM of understory saplings with a resolution of 0.05 m was generated from the normalized high-density point clouds that had completely removed the points of upper mature trees, and invalid values filling method [44] were further used to optimize the quality of the CHM, as shown in Fig. 5F.

2. Adaptive mean shift sapling segmentation. In this study, the mean shift algorithm combined with the generated CHM was used to delineate the saplings based on the 3D point clouds. Mean shift is an iteration algorithm of nonparametric kernel density estimation, which core concept is to cluster the points in the feature space and the points converge to the sample points where the density gradient is zero [45]. In particular, for a random sample point *x*_0_ in a point set {*x_i_*}, *i* = 1, …, *n*, it is iteratively updated with:

$$x_{0}\leftarrow \frac{\sum_{i=1}^{n}x_{i}K\left({\left\|\frac{x_{0}-x_{i}}{h}\right\|}^{2}\right)}{\sum_{i=1}^{n}K\left({\left\|\frac{x_{0}-x_{i}}{h}\right\|}^{2}\right)}$$ (2)

where *K*(.) is the Gaussian kernel function, which was commonly used in the mean shift algorithm and has excellent mathematical characteristics. The equation form of *K*(.) is as follows:

$$K\left({\left\|\frac{x_{0}-x_{i}}{h}\right\|}^{2}\right)=\left\{\begin{array}{cc} \exp\left(-\frac{1}{2}{\left\|\frac{x_{0}-x_{i}}{h}\right\|}^{2}\right) & \text{if}\;{\left\|\frac{x_{0}-x_{i}}{h}\right\|}^{2}\leq 1\\ 0 & \text{otherwise} \end{array}\right.$$ (3)

In Eqs. 2 and 3, *h* is the bandwidth of the Gaussian kernel function.

The traditional fixed bandwidth mean shift has been proved to be robust in individual tree segmentation [46]. However, different stem density and crown size of saplings bring challenges to obtain ideal segmentation result with a fixed kernel function bandwidth. Therefore, we proposed an adaptive bandwidth calibration method that takes into consideration spatial distribution and size of saplings calculated from the CHM.

First, the local maximum values were detected on the basis of the CHM and considered to be the reference locations of saplings. The local stem density of each reference sapling was indicated through calculating the average distance between the sapling and its nearest 4 trees. Second, because of differences in grayscale values between tree crowns and gaps on CHM, the region growing algorithm [47] was used to detect the local minimum points around each reference location and constitute the crown boundary. In addition, the circumscribed circle of each closed boundary was calculated, and its radius was regarded as another control factor of the local bandwidth. In general, local bandwidth should be proportional to the crown size of each tree and inversely proportional to the stem density. Therefore, adaptive kernel bandwidth was defined as:

$$h_{\mathrm{loc}}^{m}=\partial \times \frac{{\text{radius}}_{m}}{{\mathrm{sd}}_{m}}$$ (4)

where, $h_{\mathrm{loc}}^{m}$ is the calibrated bandwidth at the location that matching the *m*th reference sapling. *radius_m_* indicates the crown size of the *m*th reference sapling, and *sd_m_* is the local stem density around the *m*th reference sapling. *∂* is a global value, and it indicates the control factor of *radius_m_* and *sd_m_*, which can be adjusted manually to obtain the best segmentation results. The method was iteratively implemented within the study area until all sapling points were segmented.

#### Evaluation of segmentation accuracy

For each understory sapling detected from LiDAR data, tree height and position were represented by the point with the maximum height value and its corresponding plane coordinates, and crown width was the average width in north–south and east–west directions. As shown in Fig. S3B, the ALS technology generally cannot characterize the terminal leader shoot of the sapling. Therefore, the sapling height from the ALS data was treated as the vertical distance between current-year branch and ground. In contrast, as a ground-based LiDAR data acquisition technology, TLS data characterize the terminal leader shoot of the sapling well. Therefore, the phenotypic parameters calculated from TLS data were used as reference values to validate the underestimation degree of sapling height and the accuracy of crown width obtained from ALS data.

The sapling height measured in the field using visual detection method included the terminal leader shoot (Fig. S3A). According to the growth law of *P. asperata* [48], the terminal leader shoot of current year will develop lateral branches in the next year, which can be detected by the ALS technology, and new shoot will sprout out on the basis of the old terminal leader shoot. To verify this phenomenon, the point clouds of the terminal leader shoot of each sapling obtained from TLS data were removed from sapling height calculation (Fig. S3C), which would be compared with the corresponding field measurement height obtained in 2017. If they have strong correlation, the field measurement height of the whole sample plot obtained in 2017 can be used to validate the accuracy of the height detected from the ALS data of 2018.

The matching method proposed by Eysn et al. [49] was used to identify which of the upper large trees with known stem locations could be matched to the trees delineated by the NSC or NSCP method. Saplings detected from different data source were matched according to the given horizontal distance and height threshold. For each reference sapling, we searched all the segmented saplings within the distance threshold of 1 m. If no segmented saplings meet the condition, the reference data did not have a matching sapling. If some segmented saplings met the condition, we selected the sapling with the minimum height difference and within the 1.5-m height threshold as the matching sapling. Finally, the detection results were respectively assessed through the detection rate, matching rate and commission rate [31].

$$R_{\det}=\frac{N_{\det}}{N_{\mathrm{ref}}}$$ (5)

$$R_{\mathrm{mat}}=\frac{N_{\mathrm{mat}}}{N_{\mathrm{ref}}}$$ (6)

$$R_{\mathrm{com}}=\frac{N_{\mathrm{com}}}{N_{\det}}=1-\frac{R_{\mathrm{mat}}}{R_{\mathrm{ext}}}$$ (7)

where *R_det_*, *R_mat_*, and *R_com_* indicate the detection rate, matching rate, and commission rate, respectively. *N_det_* indicates the number of saplings detected through the proposed method. *N_ref_* is the number of saplings measured from the field. *N_com_* indicates the number of oversegmented saplings. *N_mat_* indicates the number of the detected saplings that matched with the measured saplings.

## Results

### Delineation result of overstory trees

Figure S4 exhibits the comparison of overstory delineation results of a tile before and after applying the postprocessing algorithm. Figure S4A shows the result of crown boundaries and individual tree positions calculated through the original NSC algorithm and superimposed on the corresponding CHM. Although this method obtains relatively accurate results of most trees, there are still some trees clustered together with all or part of the trees close to them. In comparison, higher accuracy and clear crown boundaries were completely obtained through further processed using the proposed postprocessing method, as shown in Fig. S4B. Figure S4C exhibits the top view of the final segmentation result of the corresponding tile.

The detection rate and the matching rate of the NSC algorithm are 100.88% and 88.93%, respectively. Meanwhile, these values are 110.21% and 96.69% for the NSCP method. The proposed postprocessing method improved the matching rate at the cost of slightly improving the detection rate.

Furthermore, the detection accuracy of trunk positions for upper mature trees will indirectly affect the detection rate of lower saplings. Figure S5 exhibits the comparison of trunk position errors of the trees that match the field measuring data before and after postprocessing. Overall, the improvement of crown boundary extraction has reduced the trunk position errors of the mature trees.

### Understory sapling detection

**Fig. 6.**
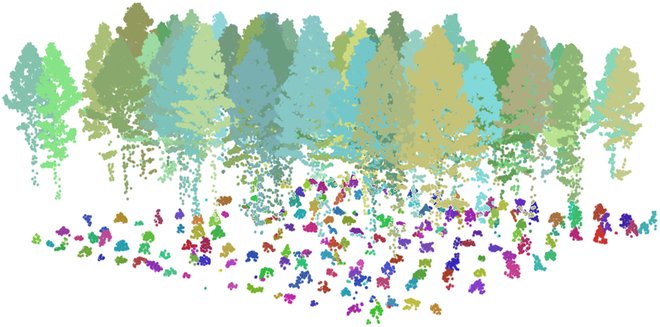
Detection results of saplings under upper mature trees. The point clouds of each upper mature tree or sapling obtained through segmentation were rendered with different colors.

On the basis of the adaptive mean shift method, the saplings were detected, and the corresponding phenotypic parameters were calculated. Figure 6 exhibits the detection results of the saplings under upper mature trees. Points with the same color represent a mature individual tree or a sapling. The optimal parameter of kernel bandwidth was determined through selecting a representative area with the size of 50 m × 50 m, and the extraction and matching rates were used to measure the availability of parameter. The result is shown in Fig. S6. Overall, when the parameter *∂* in Eq. 4 was set to a value greater than 0.5, the method can achieve a matching rate of approximately 83% with an extraction rate of 102% to 105%. Therefore, the optimal parameter of kernel bandwidth was set to 0.5 in this study to achieve sapling segmentation for the entire study area.

### Sapling phenotypic parameters evaluation using multisource reference data

**Fig. 7.**
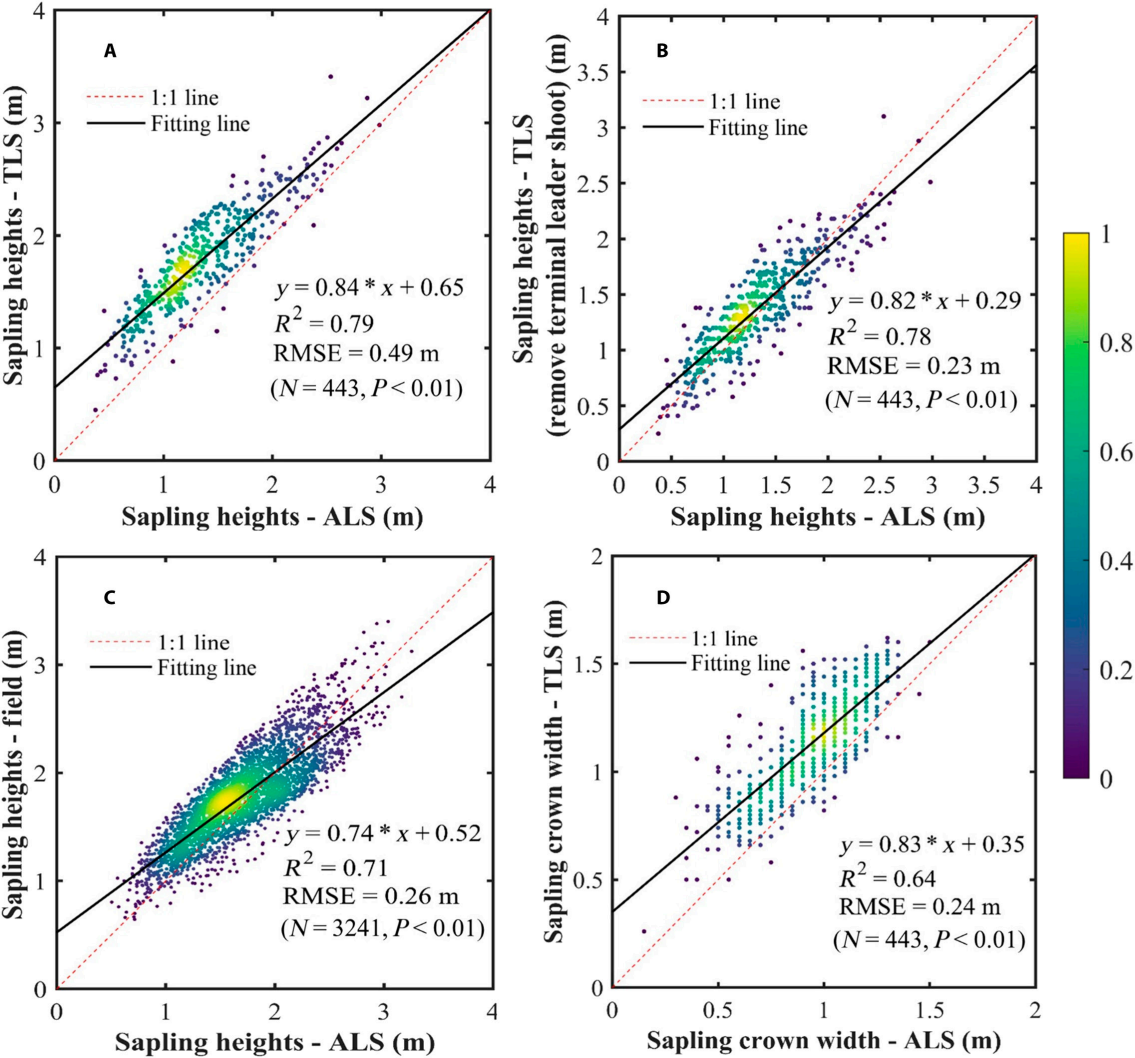
Comparisons between the sapling height estimates of ALS-based method and TLS-based method (A), TLS-based method without sapling terminal leader shoot (B), and field measurements (C). (D) The comparison between the sapling crown width estimates of ALS-based method with the ones obtained using TLS-based methods.

As shown in Fig. 7A, when compare the tree height values detected from the ALS data with the values detected from the TLS data obtained almost simultaneously, *R*^2^ of 0.79 and root mean square error (RMSE) of 0.49 m were obtained. However, it is difficult for ALS technology to fully detect the terminal leader shoot of saplings, which can be obtained by TLS. This has led to the heights of saplings being underestimated by ALS, especially for the saplings with lower heights. All the terminal leader shoot point clouds of saplings were removed from the TLS data, and the sapling heights were recalculated and compared with the results obtained by ALS data. The comparable *R*^2^ of 0.78 was obtained, while the RMSE was reducing over 50%. The linear regression result of the sapling heights calculated from the 2 data source was closer to the 1:1 line (Fig. 7B). When comparing the heights of saplings obtained by ALS in the whole sample plot with the field measured values obtained in the same month of the last year, an overall *R*^2^ of 0.71 and RMSE of 0.26 m were obtained (Fig. 7C). Because of the fact that the height of the sapling measured manually in the last year include the sapling terminal leader shoot, it indicates that the sapling terminal leader shoot had already grown lateral branches when obtaining ALS data 1 year later, which can be detected by ALS. Therefore, the ALS technology can measure the height of sapling in the previous year more accurately, and the field measured phenotypic parameters in 2017 can be used to evaluate the accuracy of sapling heights extracted using the proposed method based on the ALS data of 2018.

Because of the ability of TLS technology to obtain complete information for the understory environment, this study use the crown width of saplings obtained from TLS as reference data to measure the accuracy of ALS data in characterizing the canopy phenotypic of saplings. By matching segmentation results calculated from the point clouds obtained from different laser scanning patterns, a total of 443 corresponding saplings were obtained. Figure 7D indicates the comparison of sapling crown width estimates respectively calculated from ALS and TLS data, and the acceptable *R*^2^ of 0.64 and RMSE of 0.24 m were obtained. However, because of the obstruction of mature trees in the upper layer, the crown point clouds of most saplings obtained through ALS are incomplete, which leads to varying degrees of underestimation of the crown width of these saplings.

### Detection and matching rates of saplings

**Fig. 8.**
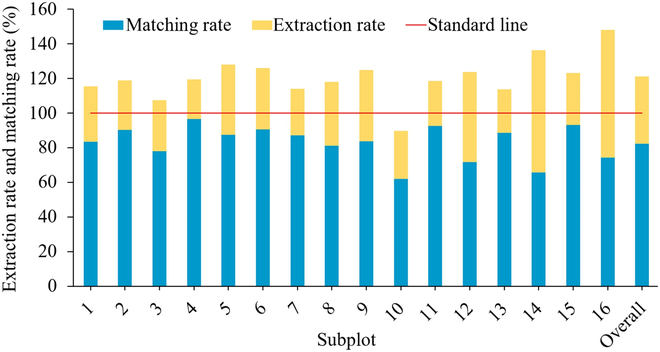
Detection and matching rates for the whole sample plot and 16 subplots.

The detection rate, matching rate, and commission rate of each subplot and the overall large sample plot were calculated according to Eqs. 5 to 7, and the results are shown in Fig. 8. The detection rates ranged from 89.76% to 147.96% with an overall value of 121.03%. The matching rates ranged from 61.89% to 96.44% with an overall value of 82.08%. The subplot 10 has the lowest detection rate (89.76%) and matching rate (61.89%), and the subplots 12, 14, and 16 have relatively high detection rates (123.67%, 136.18%, and 147.96%) with low matching rates (71.68%, 65.61%, and 74.07%) compared to other subplots. Meanwhile, the matching rates of the subplots 2, 4, 6, 7, 11, 13, and 15 are all above 88% with an average detection rate of about 91.20%, in which the subplot 4 has an extremely high matching rate of 96.44%.

### Sapling height evaluation of subplots using the field measurement data

**Fig. 9.**
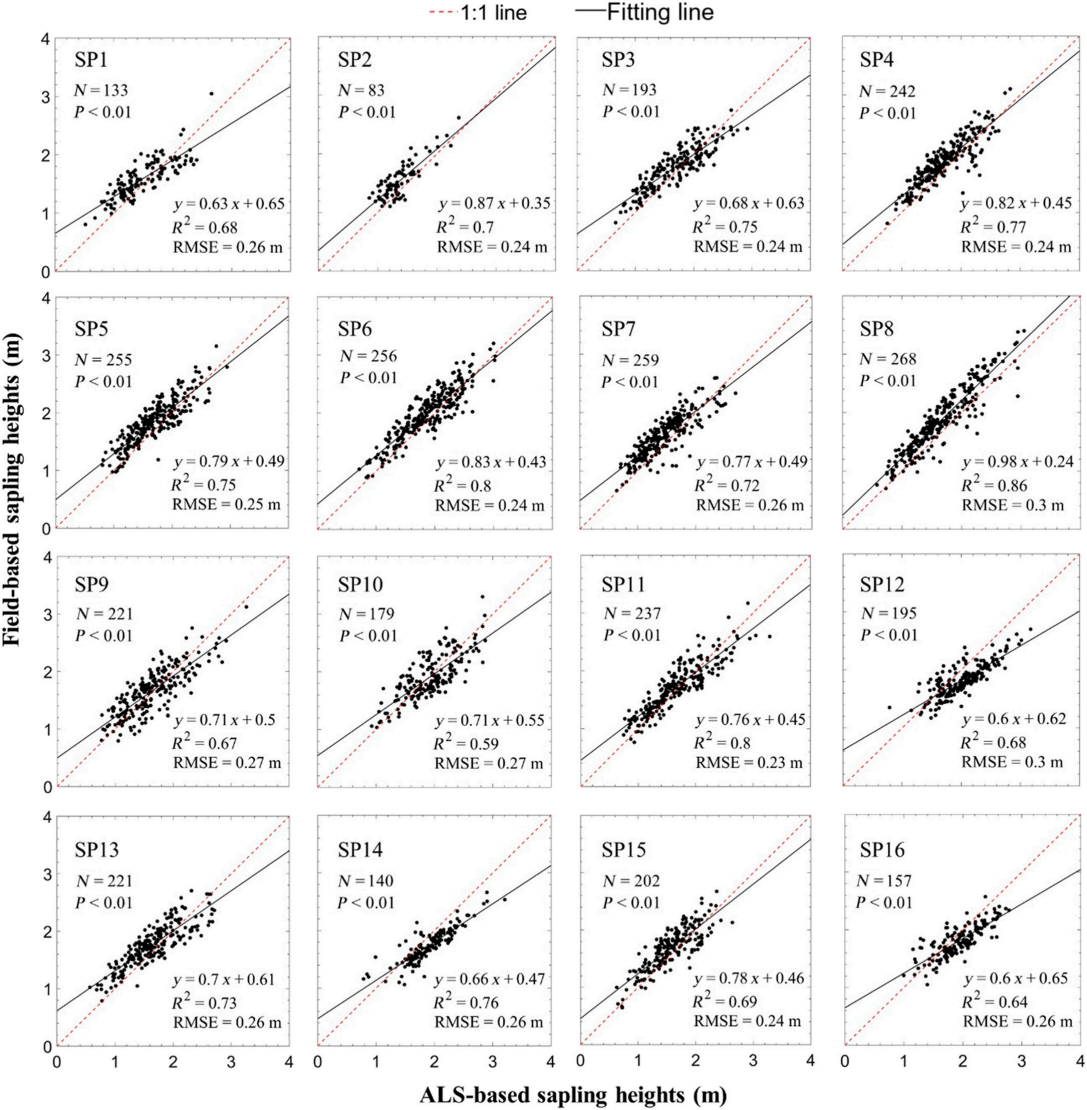
Comparisons between the ALS- and field-based understory sapling heights for 16 subplots using linear regression models.

**Fig. 10.**
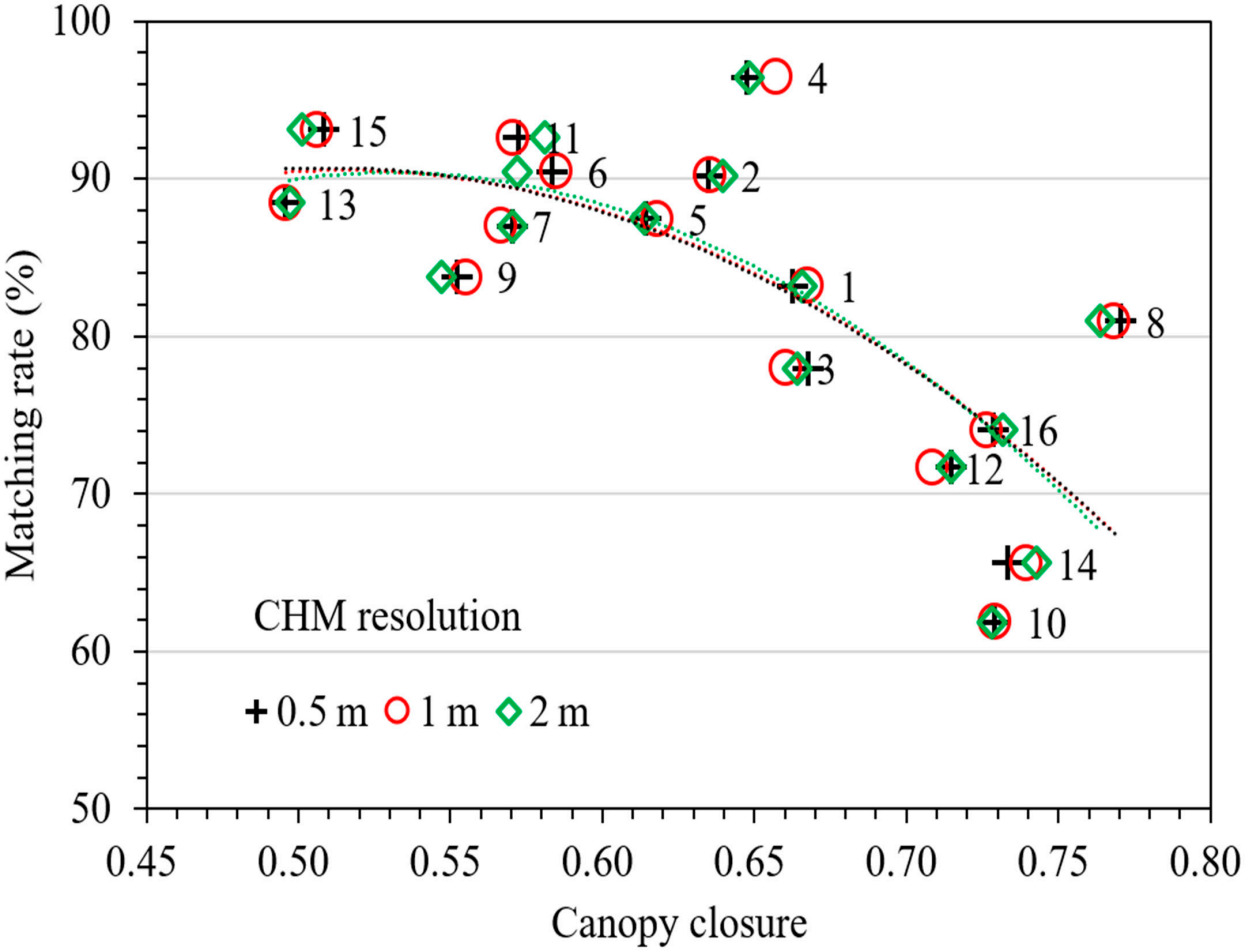
The relationship between canopy closure and matching rates for 16 subplots marked with the number of plots.

Figure 9 exhibits the tree height accuracy of all the subplots obtained from the matched saplings. Overall, the detected sapling height values were highly comparable to the height reference data. The *R*^2^ and RMSE values calculated by comparing the detected values to the reference data ranged from 0.59 to 0.86 and 0.23 to 0.3 m, respectively. In echo with Fig. 8, the subplot 10 has the lowest *R*^2^ (0.59) and relative higher RMSE (0.27 m) values, which mainly caused by the high canopy closure of the upper mature trees (as shown in Fig. 10) and the shrubs that close to the saplings in the area with large forest gap. For the same reason, the subplots 9, 12, and 16 also have relatively lower *R*^2^ (0.67, 0.68, and 0.64) and higher RMSE (0.27, 0.3, and 0.26 m) values compare to other subplots. Benefited from lower canopy closure and less shrubs and grass interference in the lower forest layer, the subplot 8 has the highest *R*^2^ (0.86) and lowest RMSE (0.23 m), and there was no significant accuracy difference within other subplots (the *R*^2^ values were in an extremely narrow range of 0.7 to 0.8, and RMSE values were close to 0.24 m).

## Discussion

Evaluating the growth of saplings under the forest and intervening in appropriate management methods in time can improve the utilization rate and productivity of the forest land [50]. With the characteristics of convenience, low labor intensity, high timeliness, and high canopy penetration, ALS has become the main technology for forest monitoring. However, most of the previous studies focused on single-layer mature or near-mature forests and young forests [51,52,53]. In this study, we explored the possibility of using high-density airborne LiDAR to detect the understory saplings and proposed a fully automated sapling segmentation and phenotypic parameter extraction scheme.

High-density ALS data can not only penetrate the canopy to obtain most of the understory sapling point clouds but also obtain relatively complete trunk point clouds of mature trees in the upper layer and mix with saplings, shrubs, and grass points [54]. Therefore, effective removal of trunk points of the upper mature trees is the prerequisite for accurate extraction of saplings. Errors in upper individual tree segmentation will lead to the estimation deviation of the tree positions [55] and then make the understory saplings close to them or planted between the undersegmented trees erroneously removed. The individual tree segmentation optimization method proposed in this study can further improve the detection accuracy of individual tree location on the basis of obtaining more accurate crown boundary (Figs. S4 and S5). On the basis of effectively removing the upper mature tree points, this study adopted a mean shift algorithm based on adaptive parameters to directly extract the position and phenotypic parameters from the sapling point clouds. This method not only takes into account the spatial distribution difference of saplings but also reduces the error caused by converting to grid and extracting trees. The field measured data were utilized to verify the results, and the average detection rate, matching rate, and commission rate were 121.03%, 82.08%, and 32.18% (Fig. 8). Most of the subplots have achieved high matching rate with appropriate detection rate. However, the commission rate of subplots 14 and 16 was relatively high (19.30% to 51.82%) compared with the other subplots, which is mainly caused by noise points of shrub and grassland under the forest. Incomplete removal of these noises leads to the algorithm recognizing them as separate saplings. Therefore, it is necessary to develop more robust and effective point cloud filtering algorithms that take into account the morphological characteristics of saplings.

Figure 7 indicate that within a 1-year interval (from August 2017 to September 2018), the height growth of spruce saplings is exactly the length of the terminal leader shoot. However, this is just a preliminary measurement result. More period data are needed if we want to obtain more accurate growth rate for this tree species. On this basis, the proposed algorithm can provide reference for the growth status of spruce saplings in the current year. Meanwhile, for the tree species of understory saplings without terminal leader shoots and can be completed detected from ALS technology, this algorithm can accurately detect their tree height in the current year. With the continuous development of ALS technology and the increase in point cloud density, there is a great possibility for detecting the terminal leader shoot of the sapling in the future.

As shown in Fig. 9, the results of sapling extraction at subplot scale show that the detection accuracy of saplings in different regions varies greatly (*R*^2^ varies from 0.59 to 0.86), which may be affected by the size of forest gap of upper canopy. Forest canopy closure refers to the extent of tree canopy covering the ground, which has a great impact on the penetration of LiDAR and can indirectly express the size of the upper forest gap [56]. Therefore, canopy closure of each subplot with different scales was used to evaluate their impact on the accuracy of understory sapling segmentation. In this paper, the proportion of the pixel area greater than 5 m to total area was calculated on the basis of the CHM and regarded as the canopy closure.

Figure 10 indicates that the matching rate of the subplots exhibits similar negative correlation with canopy closure at different scales (0.5, 1, and 2 m). In general, lower canopy closure of upper mature trees lead higher matching rate of understory sapling. Significantly, the subplots 4, 8, 10, and 14 have relatively large distance from the fitting lines compared with other subplots. Among them, the subplots 4 and 8 achieved better matching rate than predicted, but the subplots 10 and 14 achieved lower matching rate than predicted. This is mainly because there are almost no shrubs in the lower forest layers of the subplots 4 and 8, and the thickness of grassland is low, but the opposite conditions exist in the understory of subplots 10 and 14.

In this study, point cloud density is a critical factor restricting the extraction of understory saplings. In general, there is no high requirement for the point cloud density for the individual tree segmentation of upper mature trees. According to different algorithms adopted (raster-based or point-cloud-based), less than 10 points/m^2^ were adequate [57,58]. However, low point cloud density is virtually impossible to achieve the complete characterization of understory saplings. Take this study as an example, even if the point cloud density of the ALS data we used reaches 243 points/m^2^, it is still difficult to obtain the terminal leader shoot of understory saplings (Fig. 7 and Fig. S3), which has resulted in the height of most saplings being underestimated. To balance the high point cloud density and low data acquisition cost, it is necessary to further study the relationship between the penetration ability of sensors to canopy, flight angle, and flight height combined with the canopy closure of the upper mature trees to obtain point cloud data with appropriate density. In addition, there is a height difference between the mature trees and saplings in our study area. We have set a fixed height threshold of 5 m to separate the point clouds of mature trees and saplings, which reduces the computational pressure of mature trees segmentation and noise generated during the segmentation process of saplings. To generalize this assumption, the 5-m threshold can be adaptive according to the vertical strata, which can be estimated according to the height histogram in other study areas. Furthermore, because of the limitation of data acquisition, we only tested the algorithm in a larch plantation, which has relatively regular planting characteristics of mature trees and saplings (in rows and columns) in this study. The algorithm needs to be tested in more tree species and forest conditions in the future. Through extensive literature review, it can be seen that no scholars have yet applied airborne LiDAR data to extract phenotypic parameters of understory saplings, and we hope that our results can provide some inspiration.

## Conclusion

A methodological framework for extracting phenotypic parameters of understory regeneration saplings using airborne LiDAR data was proposed. High-density ALS data can penetrate the forest canopy to achieve a relatively complete description of the forest understory environment, which makes the trunk points of upper mature trees and understory saplings mixed together. We first proposed an improved algorithm for NSC individual tree segmentation and handled the undersegmentation and oversegmentation in the upper tree segmentation results, which indirectly improved the position accuracy of upper mature trees. On this basis, the trunk points of upper mature trees mixed in saplings were successfully removed, which avoided the problems like over-removed the sapling point clouds or partly over-keeped the upper mature tree point clouds because of the inaccurate detection of the upper tree positions. Furthermore, a method for extracting understory saplings based on local adaptive clustering was developed. The optimized CHM was first generated from the denoised point clouds of saplings and used to calculate the size and local stem density parameters of each sapling. The obtained parameters were used to adjust the kernel function bandwidth of the mean shift algorithm, and the cluster analysis was conducted on the point clouds of saplings to obtain the position and height of each sapling. The results were verified with field measurement data, and the overall detection rate and matching rate were 121.03% and 82.08%. The height of saplings extracted from ALS data was compared with that measured in the field at different scales, and high consistency was achieved, which obtained and overall *R*^2^ of 0.71 and RMSE of 0.26 m. Through comparing the result with the height of saplings extracted from TLS, *R*^2^ of 0.78 and RMSE of 0.23 m were obtained. The sapling crown width detected from ALS data was verified using the TLS-based measurements, and *R*^2^ of 0.64 and RMSE of 0.24 m were obtained. 

## Data Availability

The relevant code and data of this research are available at https://github.com/limingado/NSC/tree/v1.0.0 and https://github.com/limingado/Adaptive-mean-shift.
