# Supplementary material for: Identifying Regenerated Saplings by Stratifying Forest Overstory Using Airborne LiDAR Data
Source: Plant Phenomics. 2024 Feb 8;6:0145. doi: 10.34133/plantphenomics.0145 (PMC10851578; doi:10.34133/plantphenomics.0145)
Supplement: Supplementary 1 — Figs. S1 to S6 [file plantphenomics.0145.f1.zip › Supplementary Materials.docx]

# Supplementary Materials


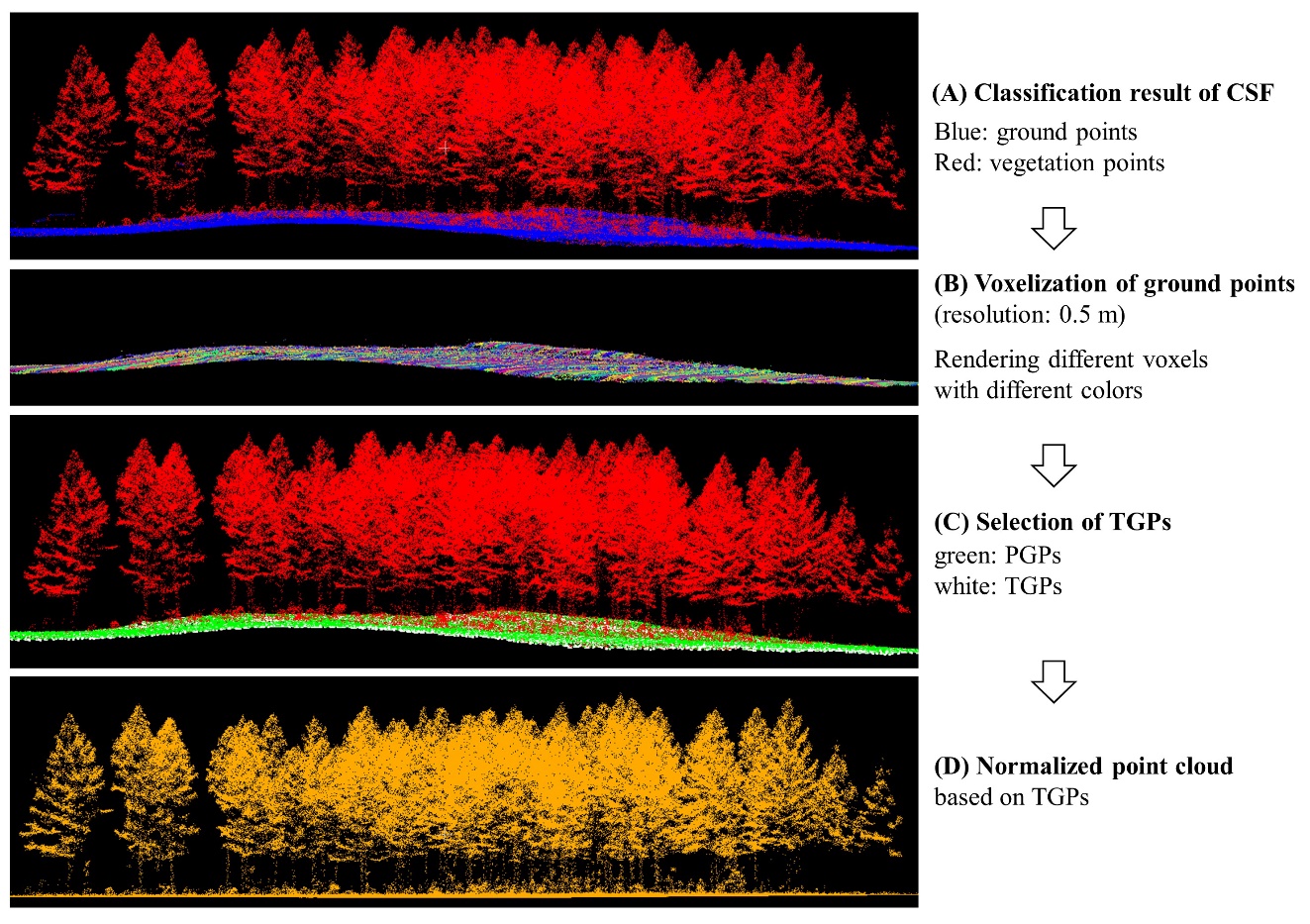


Figure S1: Fine normalization of raw point clouds. The red and blue points in (A) are respectively indicate the non-ground points and ground points obtained from CSF algorithm. The ground points in (A) were voxelized at a resolution of 0.5 m, and different colors represent different voxels, as shown in (B). The green and white points in (C) are pseudo ground points (PGPs) and true ground points (TGPs), respectively. The orange points in (D) are the normalized point clouds based on the TGPs.


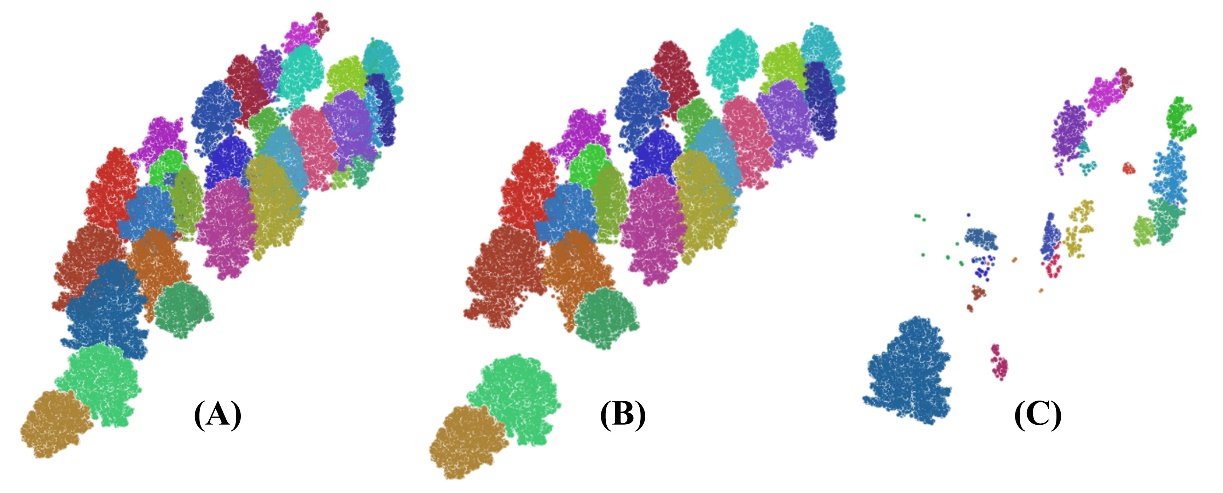


Figure S2: Selection of candidate individual trees based on control factors. (A) NSC segmentation result, (B) candidate individual trees, (C) trees containing segmentation errors.


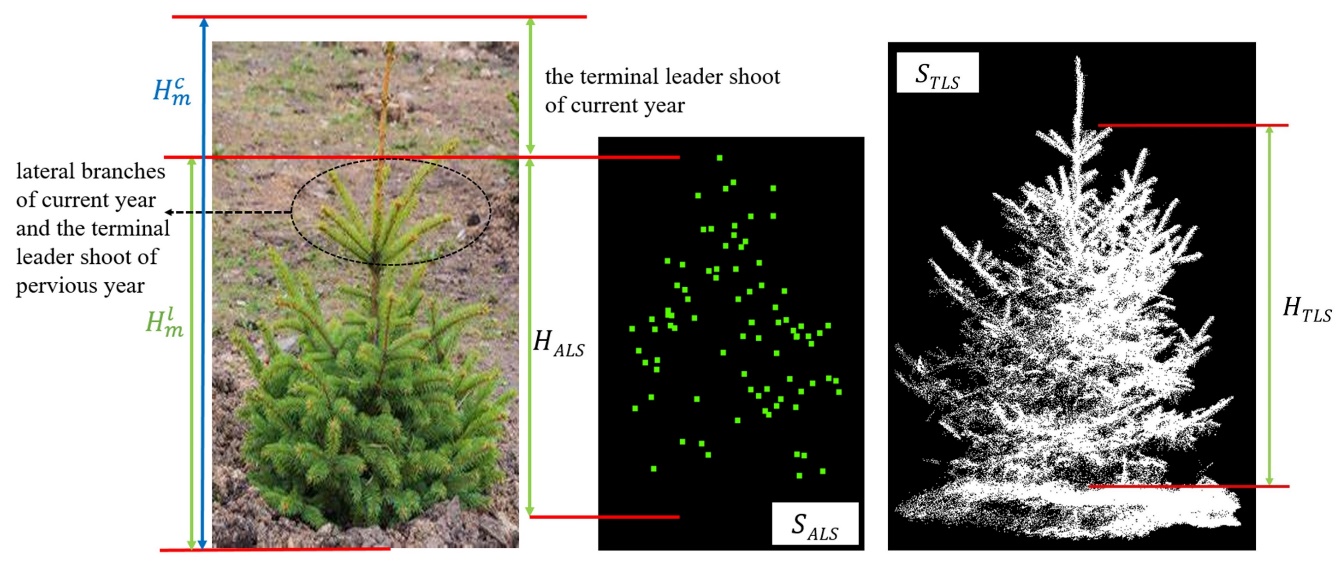


(A) (B) (C)

Figure S3: Interpretation of sapling height obtained through different technologies. (A) Sapling height obtained from field survey data, (B) sapling height obtained from ALS data, (C) sapling height obtained from TLS data.


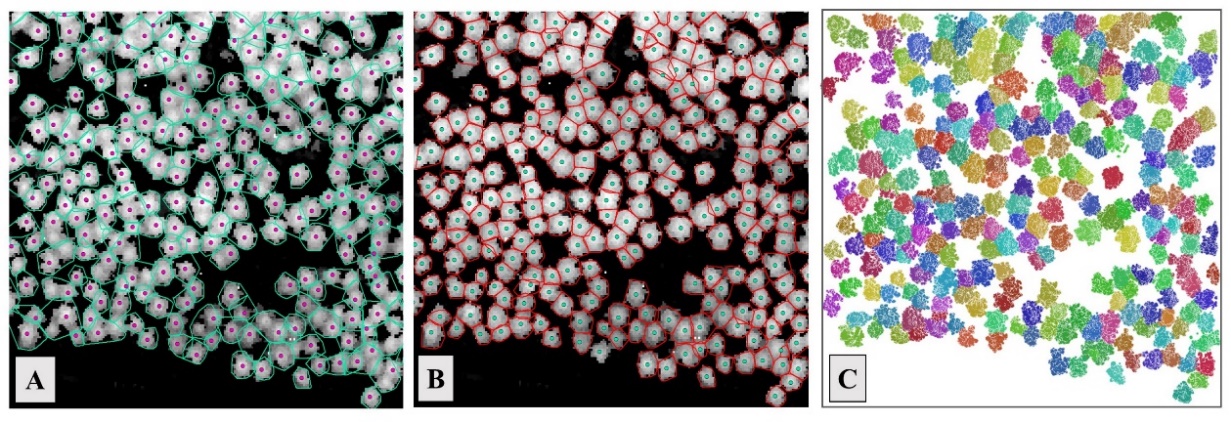


Figure S4: Segmentation result of the upper mature trees. (A) Initial segmentation results; (B) Segmentation result after post-processing, and (C) top view of the segmented trees rendered in different colors. The dots with different color in (A) and (B) exhibit the tree positions, and polygons with different colors indicate the crown boundary of each tree superimposed with CHM.


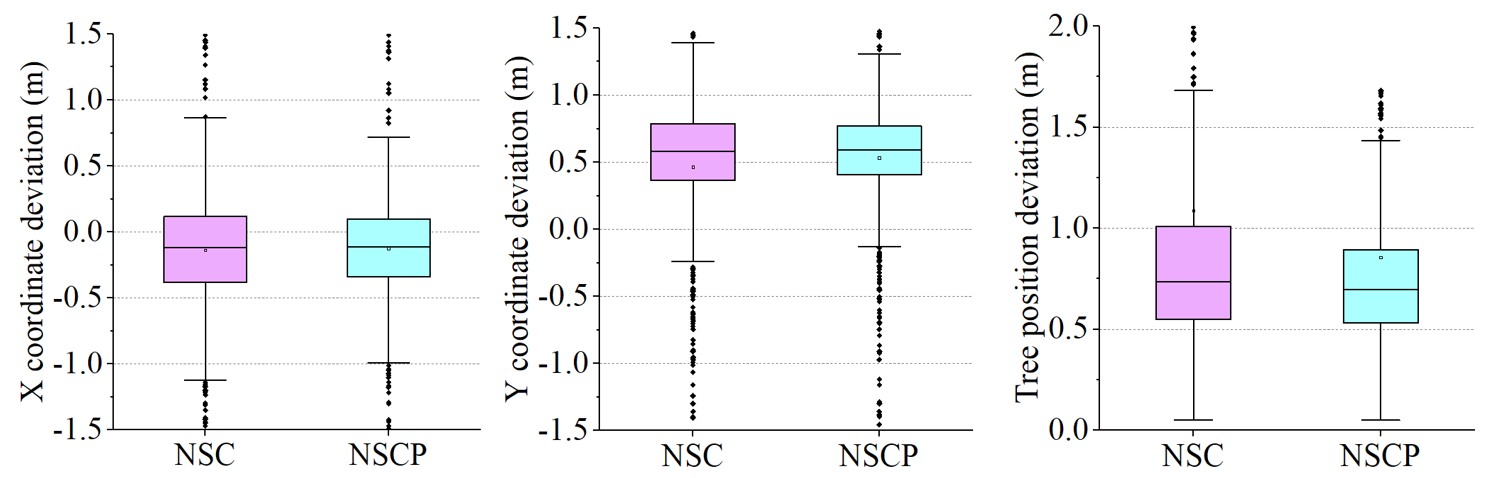


1. (b) (c)

Figure S5: Trunk position errors of the individual trees detected through the NSC and NSCP method. (a) X coordinate error; (b) Y coordinate error, and (c) trunk position error.





Figure S6: The variation of segmentation results with kernel bandwidth
