# Supplementary figures and images for: Identifying Regenerated Saplings by Stratifying Forest Overstory Using Airborne LiDAR Data
Source: Plant Phenomics. 2024 Feb 8;6:0145. doi: 10.34133/plantphenomics.0145 (PMC10851578; doi:10.34133/plantphenomics.0145)

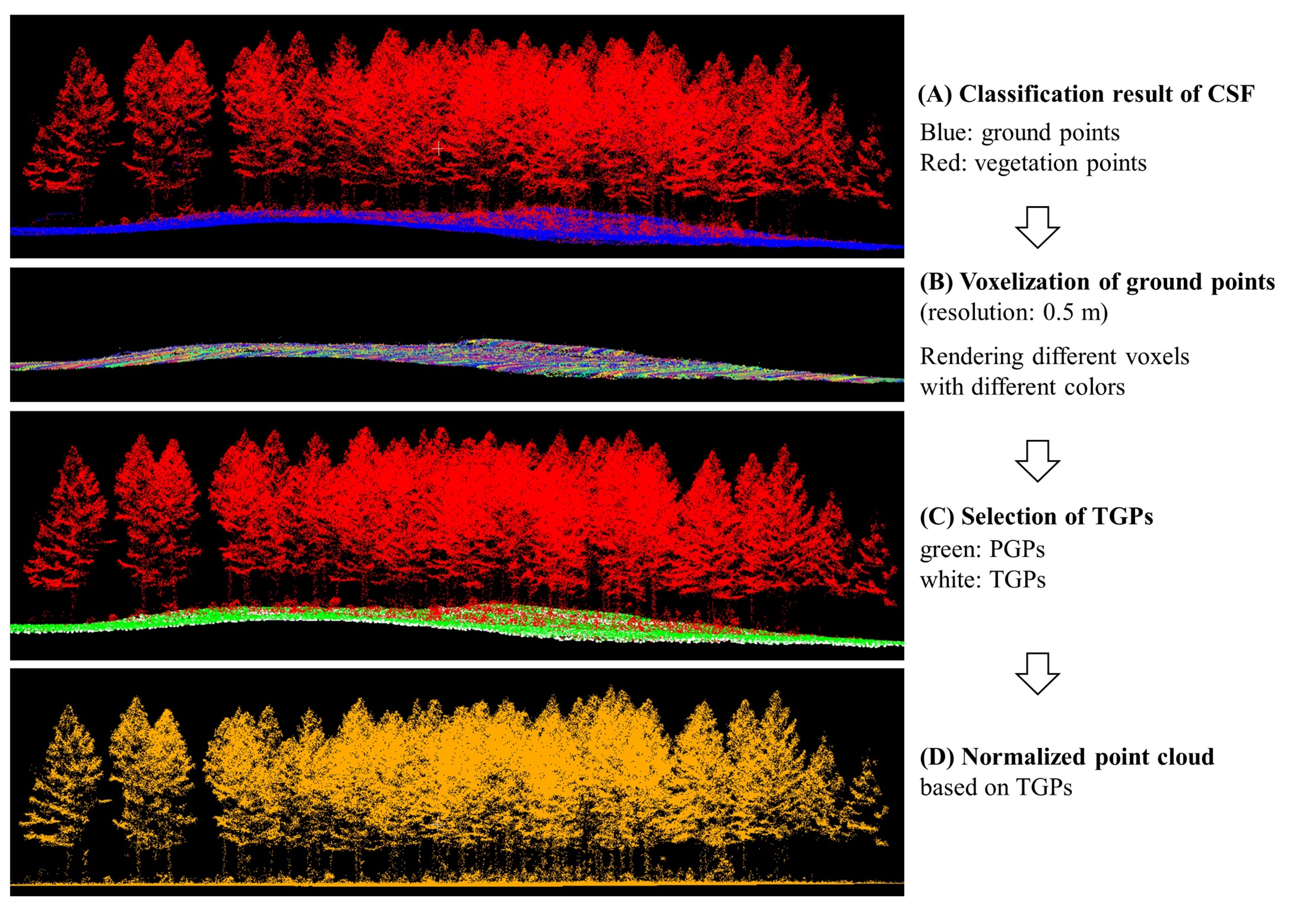

Supplement: Supplementary 1 — Figs. S1 to S6 [file plantphenomics.0145.f1.zip › Figure S1.tif]

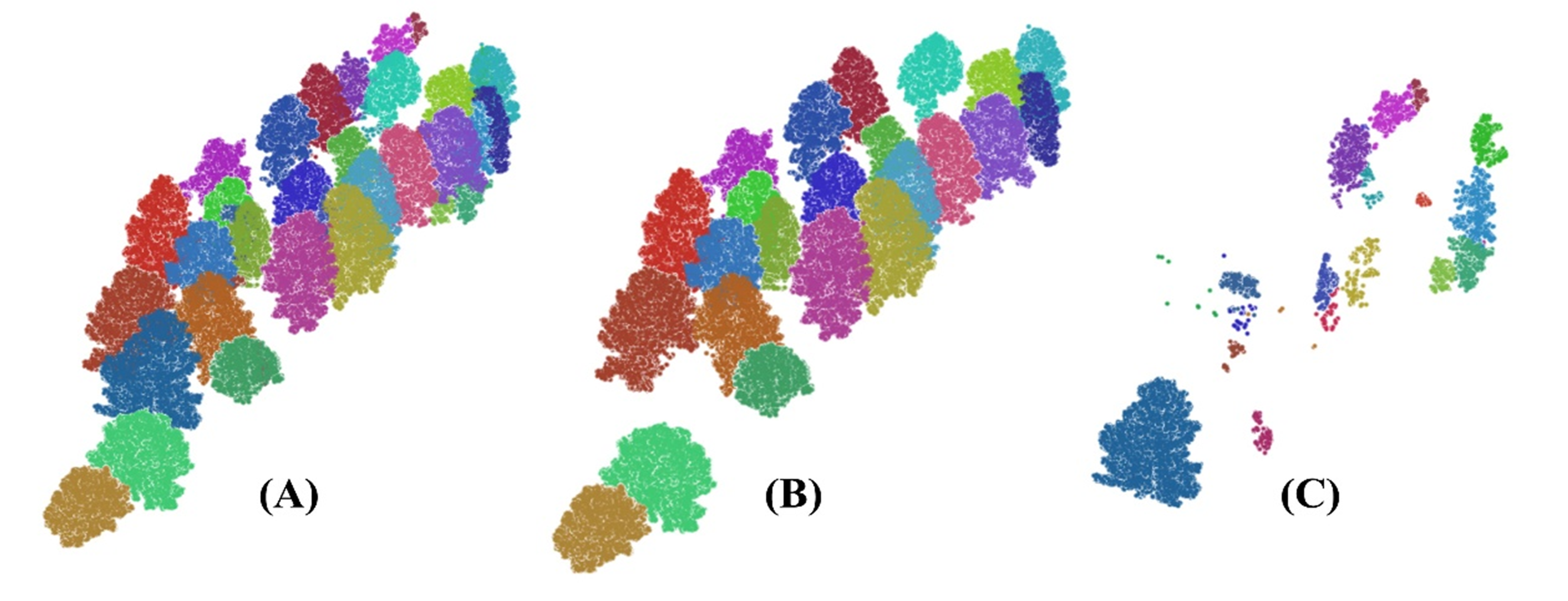

Supplement: Supplementary 1 — Figs. S1 to S6 [file plantphenomics.0145.f1.zip › Figure S2.tif]

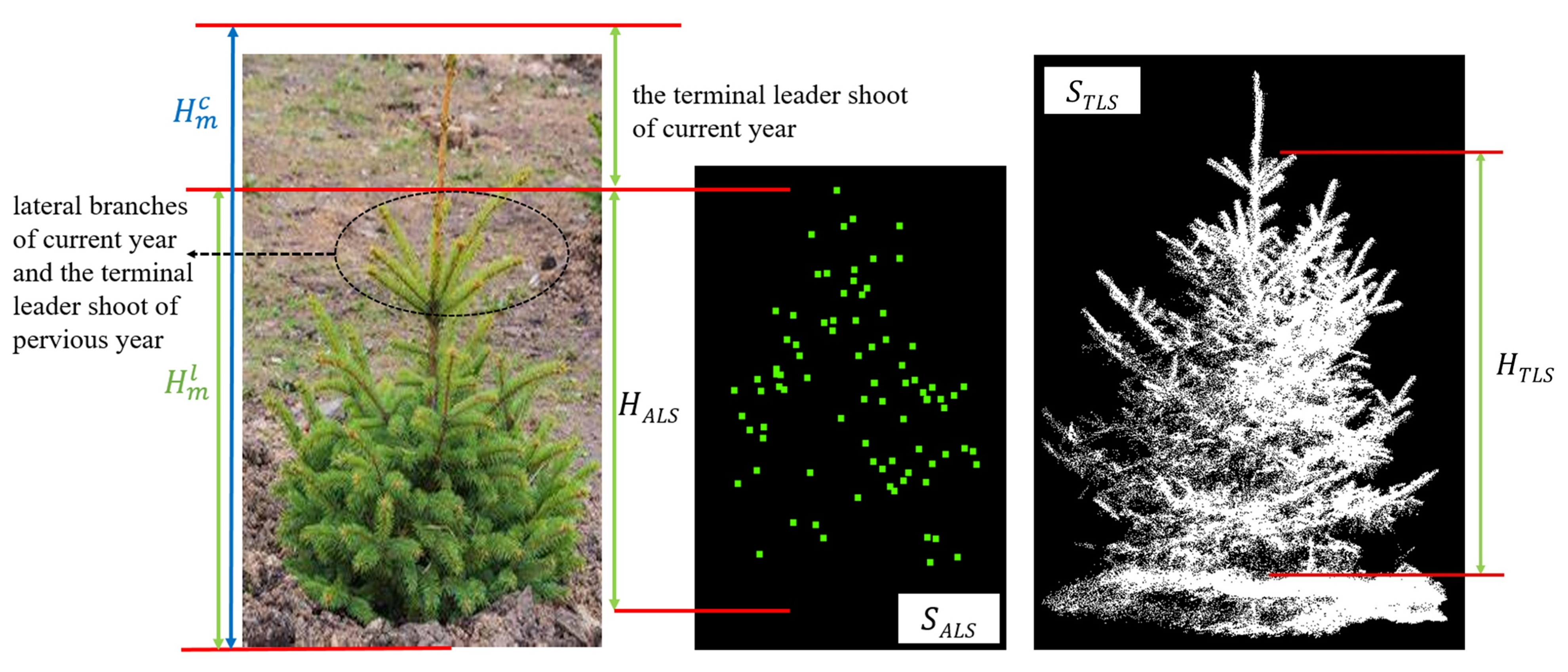

Supplement: Supplementary 1 — Figs. S1 to S6 [file plantphenomics.0145.f1.zip › Figure S3.tif]

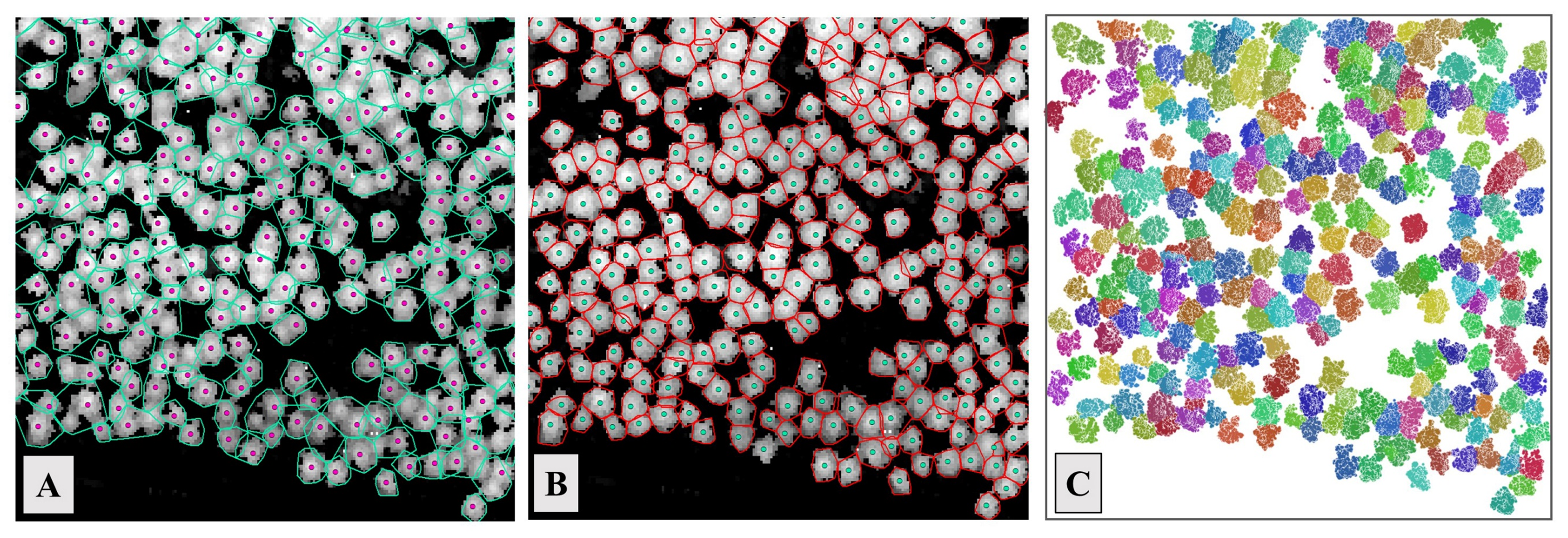

Supplement: Supplementary 1 — Figs. S1 to S6 [file plantphenomics.0145.f1.zip › Figure S4.tif]

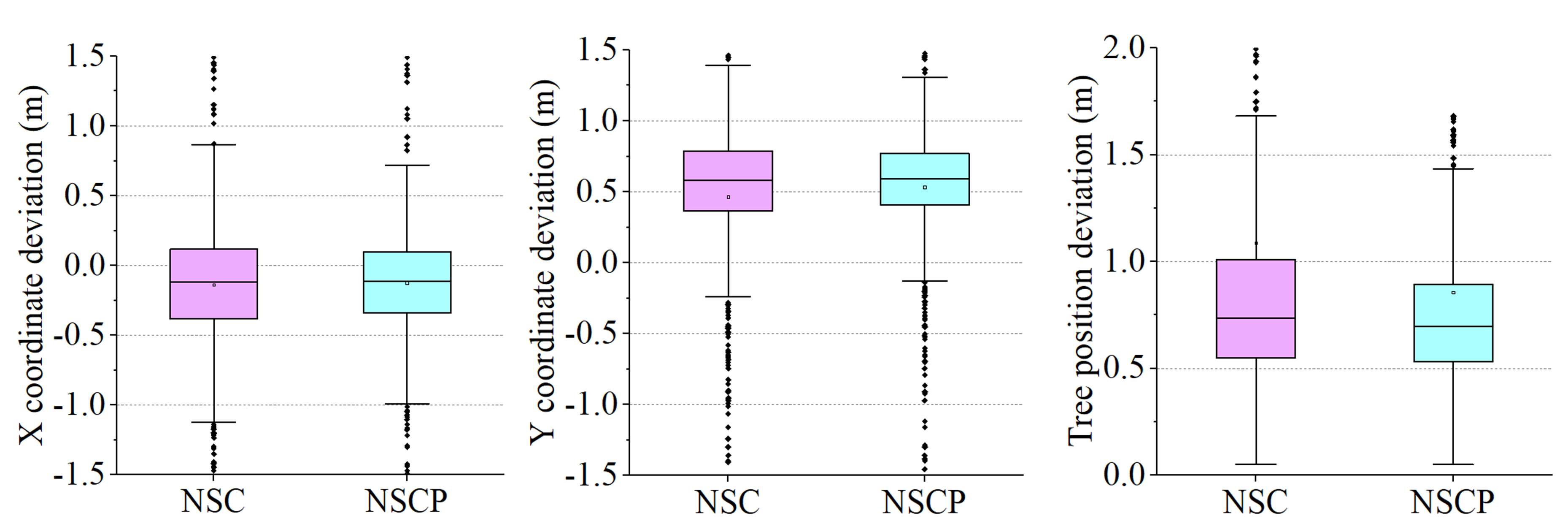

Supplement: Supplementary 1 — Figs. S1 to S6 [file plantphenomics.0145.f1.zip › Figure S5.tif]

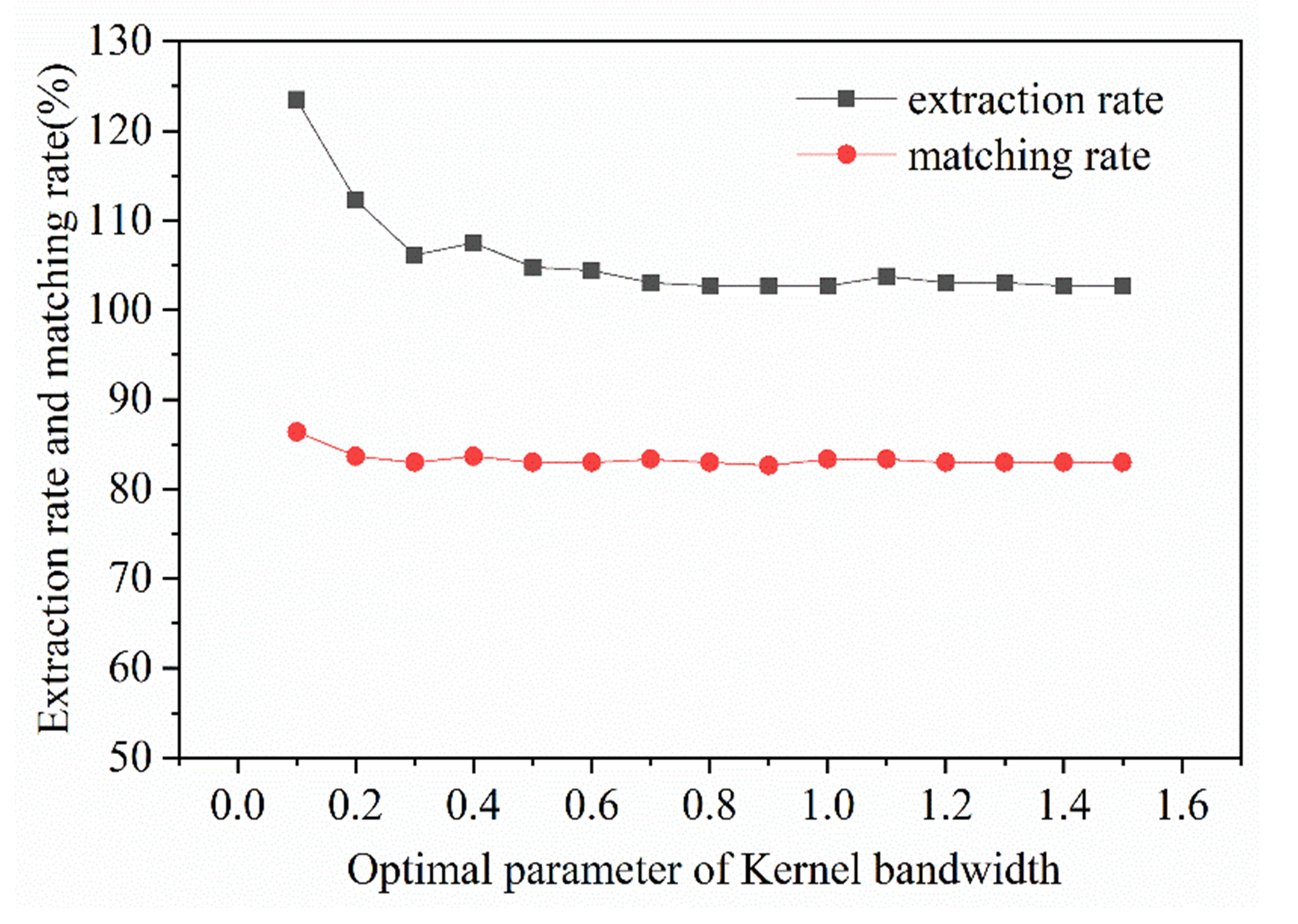

Supplement: Supplementary 1 — Figs. S1 to S6 [file plantphenomics.0145.f1.zip › Figure S6.tif]
